# *Schistosoma* infection as a cause of myeloradiculopathy: case report from Mozambique and systematic review

**DOI:** 10.3389/fmed.2026.1725323

**Published:** 2026-02-18

**Authors:** Helena Buque, Nachan Arroz, Elder Lorenzo, Alice Manjate, Alfeu Passanduca, Mohsin Sidat, Hipólito Nzwalo

**Affiliations:** 1 Department of Neurology, Maputo Central Hospital, Maputo, Mozambique; 2 Ageing and Cerebrovascular Research Group, ABC Research Institute, Faculty of Medicine and Biomedical Sciences, University of Algarve, Faro, Portugal; 3 Department of Microbiology, Faculty of Medicine, Eduardo Mondlane University, Maputo, Mozambique; 4 Department of Community Health, Faculty of Medicine, Eduardo Mondlane University, Maputo, Mozambique

**Keywords:** myelopathy, neuro-schistosomiasis, neuroschistosomiasis Mozambique, schistosomal myeloradiculopathy, schistosomal paraparesis

## Abstract

**Background:**

Schistosomal myelopathy (SM) is a rare and devastating neurological manifestation of schistosomiasis, predominantly affecting individuals in endemic regions. SM may present a wide range of positive and negative manifestations such as paresthesia, myalgia, low back pain, paraplegia and urinary dysfunction. Limited awareness and lack of access to supportive microbiological and imaging diagnostics means may delay clinical recognition.

**Methods:**

Case report of SM and a systematic review of the literature on its epidemiological patterns and clinico-radiological characteristics, including cases published from inception to April 27, 2025.

**Results:**

A 27-years-old man presented with chronic back pain, progressive paraparesis - modified Rankin 4, and urinary incontinence. Magnetic resonance imaging (MRI) revealed findings suggestive of inflammatory myelitis from T12 to L4, the cerebrospinal fluid (CSF) microscopy demonstrated the presence of *Schistosoma* eggs. The literature review identified additional 58 cases of SM. The median age was 25 years (range 2–65 years); most patients were males (79%), presenting with subacute and chronic disease. In the majority of cases, SM lesions involved the thoracolumbar region or conus medullaris. *Schistosoma* eggs were detected in 31% (*n* = 37) of cases. Additional systemic manifestations, for instance bladder involvement, were reported in 64% (*n* = 75). All patients received anthelmintic therapy and corticosteroids. At the end of follow up, 90% (*n* = 107) were able to walk unassisted, while 10% remained dependent (modified Rankin scale ≥ 3).

**Conclusion:**

Schistosomal myelopathy is a rare, aggressive neurological disorder primarily affecting young males. Even with appropriate medical therapy, a substantial proportion of patients remain functionally dependent (mRS ≥ 3). This emphasizes the critical importance of early recognition and prompt intervention to mitigate irreversible neurological impairment.

## 1 Introduction

Schistosomiasis is a highly prevalent parasitic disease caused by *Schistosoma* species, endemic in roughly 78 countries and affecting millions of individuals globally, most notably across Africa, South America, and parts of Asia. While the infection often manifests with mild or non-specific symptoms, it can progress to severe and disabling disease (1–3). It is a neglected tropical infection, acquired primarily through contact with infested freshwater or ingestion of contaminated food (4). The transmission of schistosomiasis is shaped by biological, environmental, behavioral, and socioeconomic factors (5).

Despite the evidence of decreasing disease burden from 1990 to 2021, with declining prevalence, mortality and disability-adjusted life-years (DALYs) related to schistosomiasis in Africa (3), the disease remains an important public health problem in the continent. In the Sub-Saharan Africa (SSA) region, for instance, there are approximately 192 million people infected and the mortality rate by gastrointestinal and urinary complications is estimated to be about 130 000 and 150 000 per year (6, 7). Mozambique is one of the top five SSA countries with the highest prevalence, with 13 million people with schistosomiasis (6).

Although schistosomiasis predominantly affects the gastrointestinal and genitourinary systems, ectopic deposition of *Schistosoma* eggs may lead to severe neurological manifestations, most notably schistosomal myelopathy (SM). Of the five *Schistosoma* species known to infect humans, *S. mansoni*, *S. haematobium*, and *S. japonicum* demonstrate a higher affinity for the central nervous system (CNS) (8). In addition to spinal involvement, two principal clinical forms of neuroschistosomiasis have been described: acute schistosomal encephalopathy and pseudotumoral encephalic schistosomiasis (9, 10). SM is more frequently associated with a chronic, disabling course, typically manifesting as paraparesis accompanied by urinary voiding disturbances (11, 12). Occasionally, when the cervical segment is affected, patients may present with tetraparesis (13). This condition is often an underrecognized cause of non-compressive myelopathy and is frequently misdiagnosed due to community and clinician’s inexperience (4). In the largest published case series of schistosomal myelopathy (SM), comprising 63 patients from Brazil (14), the disease most commonly presented as an acute lower spinal cord syndrome with motor, sensory, and autonomic deficits. Inflammatory changes were consistently observed in cerebrospinal fluid and neuroimaging studies, parasite loads were generally low, and approximately 60% of patients achieved favorable outcomes (14).

These patterns probably reflect regional particularities such as sociodemographic characteristics, exposure profiles, diagnostic delays, and the predominant *Schistosoma* species involved. To address this, we performed a systematic review of the literature to summarize existing evidence on the sociodemographic, clinical, and imaging characteristics of schistosomal myelopathy (SM), and we also report a representative case from Mozambique.

## 2 Methods

We present a detailed clinical, microbiological, and imaging description of a case of schistosomal myelopathy (SM) from Mozambique, alongside a systematic review of the literature. This systematic review followed the Preferred Reporting Items for Systematic Reviews and Meta-Analyses (PRISMA) guidelines. A comprehensive search of the PubMed and Scopus databases was performed from inception through April 27, 2025, using the keywords “Neuroschistosomiasis” and “CNS *Schistosoma*.” We included case reports and case series providing information on at least one of the following domains: clinical presentation, imaging findings, or prognostic factors. Studies of all ages with abstracts published in English and Portuguese were considered. We excluded abstracts or studies with unclear diagnoses, cases mimicking schistosomal infection, or those lacking evidence of medullary involvement. Two authors independently screened abstracts obtained from the database research. Duplication screening and discrepancies were evaluated and resolved by principal investigator (HB). The full text of potentially relevant articles was retrieved for further consideration. The study selection process and final number of included cases are summarized in the PRISMA flow diagram^1^.

## 3 Results

### 3.1 Case presentation

**FIGURE 1:**
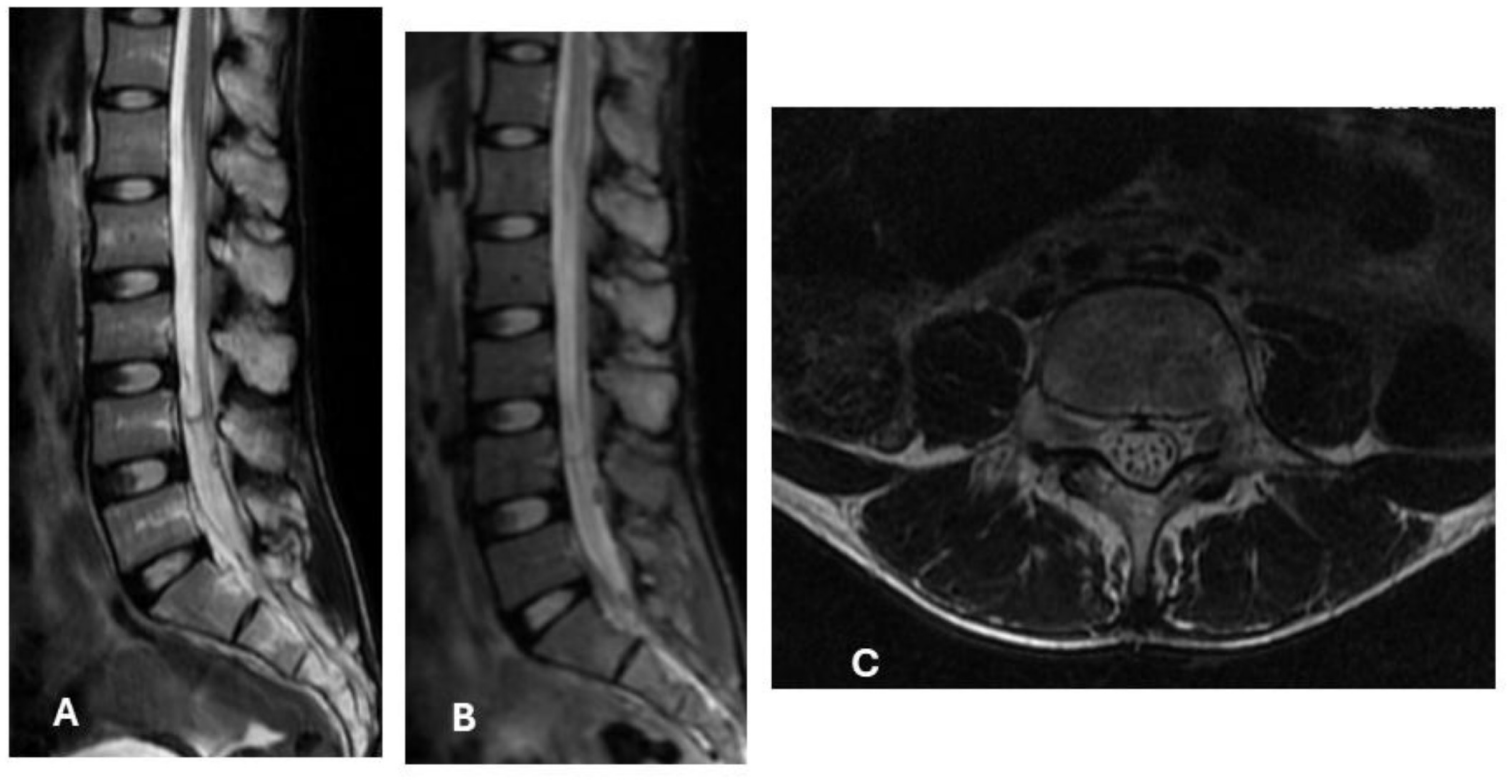
Sagittal section in T2 MRI showing intramedullary hyperintensity between Lumbar segments L1 to L4 **(A)**, and enhancing mass at the conus medullaris with extensive spinal cord edema **(B)**. Intramedullary nodular lesions exhibiting T2 hyperintensity, associated with mild spinal cord enlargement, findings suggestive of schistosomal granulomas **(C)**.

A 27-years-old male, resident in endemic area in southern region of Mozambique, presented with a progressive history of lower back pain, lower limb weakness and urinary incontinence for the last 6 years. Although the disease progressed very slowly, the initial presentation was characterized by sudden-onset of back-pain, paraparesis. Apart from urological surgery for urethral fistula correction at age 21, his past medical history was unremarkable. Family history was non-contributory, and no relevant genetic information was available. Psychosocially, the patient reported significant impact on mobility and daily activities due to chronic neurological deficits. Previous interventions included general supportive care and multiple non-specific consultations, which did not lead to diagnosis or improvement prior to referral to our neurology service. The neurological examination revealed the presence of paraparesis with bilateral lower limb weakness of grade 2 on Medical Research Council, hyperreflexia and sensory level at the thoracolumbar (T12–L4) spinal level. Laboratory findings revealed normocytic moderate anemia, and eosinophilic pleocytosis. The urethrocystoscopy revealed schematic rings at the bulbar urethra, of grade 3 strain bladder, diverticula in the posterior wall, and the presence of *Schistosoma* spp. eggs. The magnetic resonance (MRI) of the spine showed hyperintensity on T2-weighted sequences in the thoracolumbar spinal cord, with mild enhancement, diffusely enhancing mass at the conus medullaris with extensive spinal cord edema suggestive of an inflammatory/infectious etiology, as shown in Figure 1.

**FIGURE 2:**
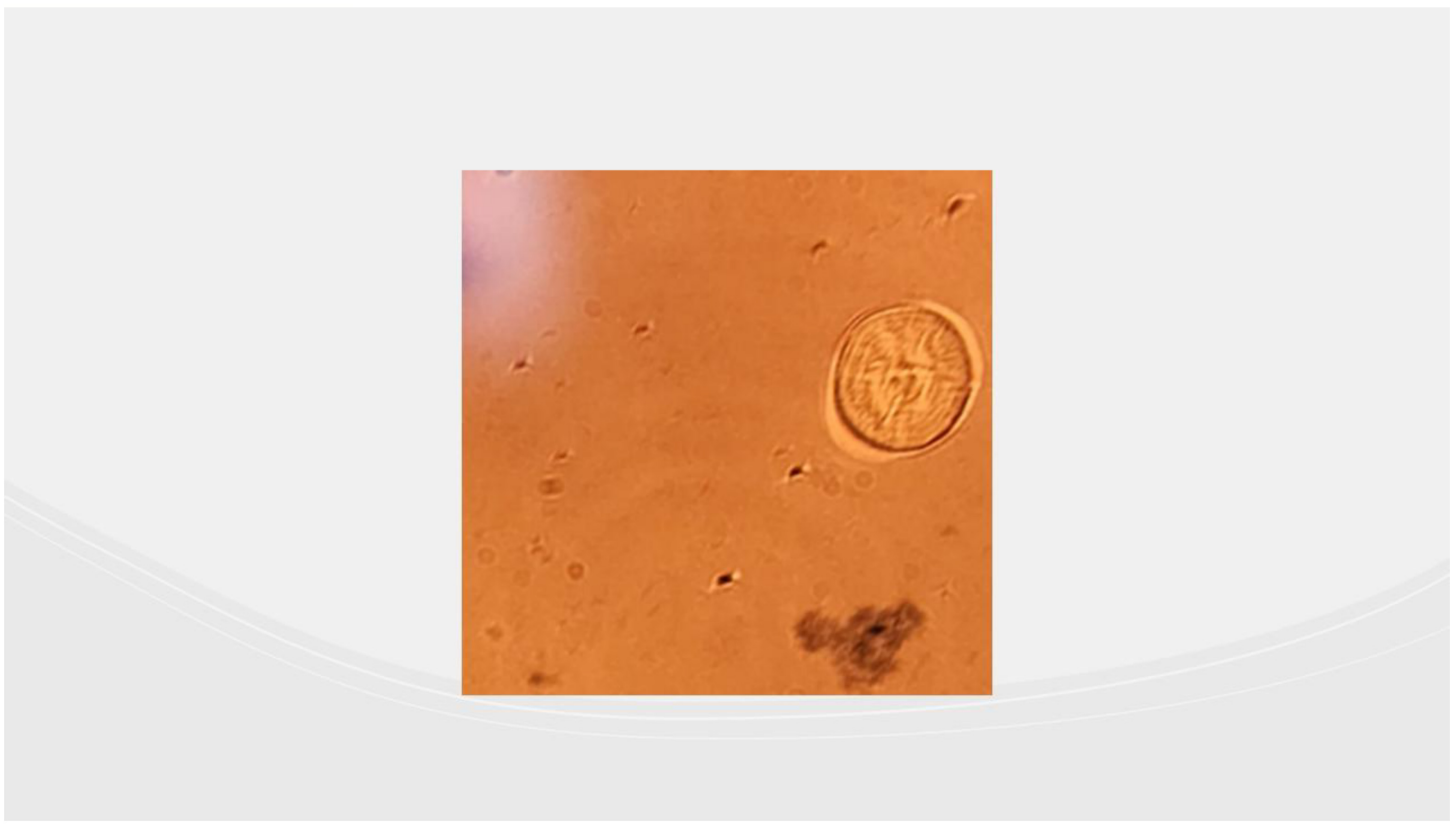
Showing CSF microscopy with one oval-shaped, and less elongated *Schistosoma*, possibly japonicum egg.

The presence of *Schistosoma* spp. eggs were detected in the cerebrospinal (CSF) microscopy, confirming the diagnosis of SM. Microscopy images demonstrated characteristic *Schistosoma* spp. eggs without lateral spines, with strong suspicion of *Schistosoma Japonicum* infection, as shown in Figure 2.

The patient was treated with oral antiparasitic therapy using praziquantel, administered according to standard dosing recommendations for schistosomiasis. Corticosteroid therapy was initiated as adjunctive treatment to reduce inflammatory spinal cord damage and edema. The choice of therapy was guided by parasitological confirmation, radiological findings, and published recommendations for the management of spinal schistosomiasis. No changes to the therapeutic regimen were required during treatment, as the patient tolerated the intervention without significant adverse effects.

**FIGURE 3:**
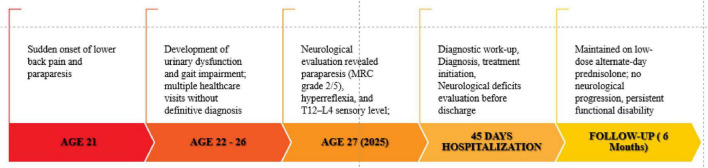
Time-line of clinical evolution, from onset of clinical manifestations to the last follow-up appointment.

Clinical follow-up included serial neurological examinations and assessment of functional status. Outcomes were evaluated using clinician-assessed motor strength, sensory function, sphincter control, and patient-reported functional ability. Although partial stabilization of neurological deficits was achieved, significant motor impairment and urinary dysfunction persisted, consistent with chronic spinal cord injury following delayed diagnosis, as shown in Figure 3.

### 3.2 Systematic literature review

**FIGURE 4:**
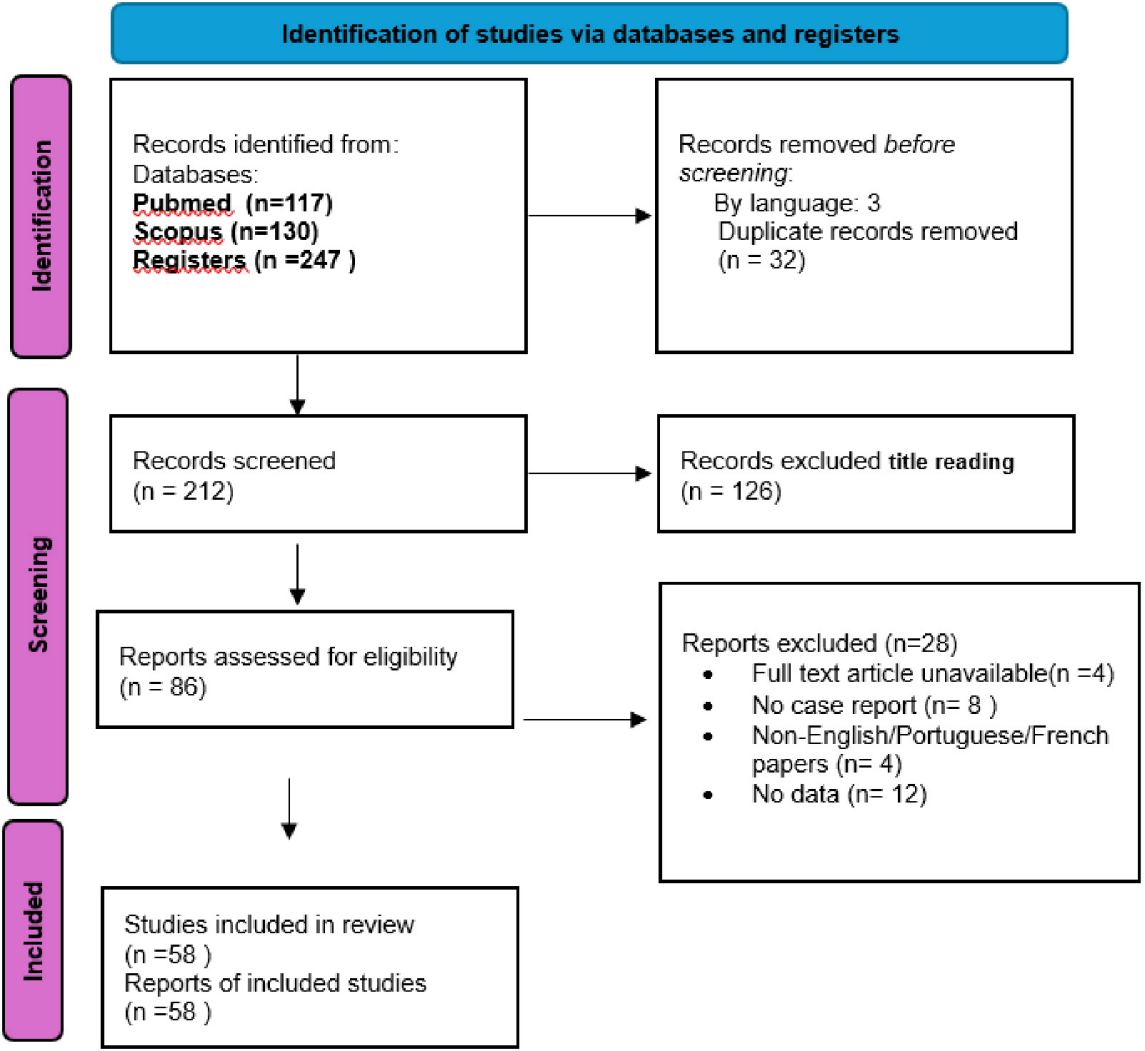
Preferred Reporting Items for Systematic Reviews and Meta-Analyses (PRISMA) flow diagram of selection and inclusion of studies in the systematic review.

Two hundred and twelve manuscripts were reviewed and 58 were included in the revision (Figure 4). The main reasons for exclusion were lack of documented spinal involvement and studies not published in English or Portuguese. Supplementary Table 1 summarizes the sociodemographic, clinical and imaging findings of SM cases. Most cases (*n* = 92, 78%) originated from low-income countries, including Ethiopia, Mauritania, South Sudan, Cameron, Senegal, Egypt, Kenya, China, Nigeria, Uganda, Mali and Eritrea. In some cases, (*n* = 16, 14%), however, the diagnosis was made in Europe, even though the disease had originated and first manifested in Africa. Ages ranged from 2 to 65 years. Among the cases with individually reported ages (*N* = 62), the median was 25 years and most were males (*n* = 93, 79%). The range time to diagnosis was 1 month to 1 year. Lesions were mostly found in thoracolumbar and conus medullaris (*n* = 58, 60%) or conus medularis isolated (*n* = 17, 18%). The most common species were mansoni (*n* = 62, 53%), haematobium (*n* = 3, 2%), japonicum (*n* = 4, 3%). Patients were treated with praziquantel (*n* = 116, 98%), oxamniquine (*n* = 2, 2%) and steroids (*n* = 118, 100%). In the majority of patients (*n* = 107, 90%), a favorable clinical course was observed, with functional independence achieved (modified Rankin score ≤ 2). Conversely, in approximately 10% of cases, recovery was not accomplished, and patients persisted with moderate to severe disability (modified Rankin score 3–5).

## 4 Discussion

In this manuscript, we describe the epidemiological patterns and clinico-radiological characteristics of SM based on published cases. The majority of cases involved patients from endemic sub-Saharan African (SSA) countries (55%) and Brazil (35%). Notably, among the African cases, 24% were diagnosed while the patients were residing in Europe. This striking finding highlights the potential for underdiagnoses or delayed diagnosis of SM, during which neurological damage may accumulate before the disease is recognized. Our case, involving a male patient with disease onset at age 21, aligns with the predominant sociodemographic profile identified in the systematic review, in which most patients are young males (79%), ranging in age from 2 to 65 years. SM affecting the lower thoracic and upper lumbar spinal cord, as observed in our patient, is a common presentation (15–40) and merits attention. The pathophysiology of SM involves the deposition of *Schistosoma* spp. eggs in the spinal cord vasculature, triggering an inflammatory response, granuloma formation, and subsequent neuronal damage (6, 41). The conus medullaris and lumbosacral region receive their blood supply primarily from the anterior spinal artery via the artery of Adamkieicz, which most commonly originates between T9 and L2 (42). One can speculate that the terminal vascularization, with fewer collateral vessels, favors the deposition and retention of *Schistosoma* eggs when the parasite reaches the area through the venous circulation. The conus medullaris, which is also commonly affected in SM, and sometime in isolation (32, 33, 43–54). The anatomical arrangement of conus medullaris which is permissive to retrograde venous reflux from the mesenteric and pelvic venous networks (55) may allow *Schistosoma* eggs and adult forms to reach the region. Consistent with this possibility, our patient also presented with a concomitant chronic urological *Schistosoma* infection. The prolonged diagnosis delay may explain this finding as time allows the inflammation and/or the infection to expand within the spinal cord. For instance, in our case, it took 6 years from disease onset to the diagnosis.

The diagnosis is based on a combination of clinical presentation, neuroimaging, and laboratory findings. The CSF analysis may show eosinophilia, increased protein concentration, and, in rare cases, *Schistosoma* spp. eggs (56). A definitive diagnosis can be established through histopathological analysis (2). The MRI typically reveals signal hyperintensity on T2-weighted images, enlargement of the spinal cord, and thickening of the spinal roots (2).

In our patient, diagnosis was confirmed by eggs of *Schistosoma* spp in the CSF, and radiologic findings, which shows medullar and cauda equina involvement and contributes for the severity and disabling sequelae.

The diagnostic delay, which we have previously demonstrated in our setting (57), remains an extremely relevant issue. In our case, the interval between symptom onset and diagnosis was 6 years, likely contributing to extensive spinal cord involvement and severe, disabling sequelae. This observation is consistent with previous reports showing that early diagnosis and prompt treatment are associated with better neurological recovery, whereas delayed diagnosis often results in irreversible deficits. The contrast between early and late diagnosis highlights the importance of timely recognition, particularly in resource-limited settings (11, 16, 19, 20, 58–64). The lack of access to appropriate CNS imaging is a reality in most sub-Saharan African countries. In this context, alternative and pragmatic clinical approaches should be considered. For patients presenting with a clinical medullary syndrome who originate from endemic areas, the detection of *Schistosoma* in the CSF may serve as a valuable indicator to initiate appropriate treatment.

Nevertheless, the presumable diagnosis can be made based on the following criteria: the presence of neurological symptoms affecting the lower thoracic and upper lumbar regions; epidemiological evidence supporting exposure to schistosomiasis; confirmation of schistosomiasis exposure through parasitological or serological testing; and the exclusion of other recognized causes of transverse myelitis and myeloradiculitis (65).

There is a classification system for assessing the probability of spinal schistosomiasis, which does not incorporate cerebrospinal fluid CSF abnormalities or imaging findings. This case classification uses different criteria as follows: Possible: presence of lower thoracic, lumbar, or sacral spinal cord involvement, combined with epidemiological evidence of schistosomiasis exposure; Probable: in addition to the above criteria, confirmation of schistosomal infection through parasitological methods and exclusion of other potential etiologies. Confirmed: histopathological demonstration of *Schistosoma* spp eggs or adult worms affecting any part of the central nervous system (56, 58, 65). In our case, this pattern is consistent with established diagnostic classification frameworks for SM, which allow for presumptive diagnosis based on compatible neurological syndrome, epidemiological exposure, parasitological or serological evidence of schistosomiasis, and exclusion of alternative causes of transverse myelitis or myeloradiculitis. Such frameworks are particularly valuable in settings where access to advanced imaging or biopsy is limited.

A pediatric case from Ethiopia described by Arega et al. highlighted spinal schistosomiasis mimicking a spinal cord tumor, with similar MRI features and diagnostic uncertainty. This case strengthens the regional perspective and reinforces the need to consider SM in the differential diagnosis of spinal cord masses in endemic areas (66).

Most cases of SM were from mansoni, haematobium and japonicum species. In SSA the most common species found was mansoni.

Treatment often involves a combination of antiparasitic therapy, such as praziquantel, and corticosteroids to eliminate the parasite and reduce inflammation. In endemic regions, early empirical treatment should be considered in suspected cases. Early intervention is crucial to prevent permanent neurological deficits (2, 67). In the reported case, as in many others from the literature (16, 19, 20, 58–64, 67), the patient was treated with praziquantel and corticosteroids, with minimal improvement observed along the time in motor function, as well as in neurological and urological symptoms. The poor response to treatment may be associated with the late diagnosis leading to permanent neurological lesions.

The patient described the prolonged diagnostic process and its impact on daily life, particularly persistent motor impairment and urinary dysfunction. Despite adherence to treatment, the patient experienced minimal functional improvement and highlighted the challenges of coping with chronic neurological disability. Including this perspective emphasizes the human impact of delayed diagnosis and the importance of timely recognition and management.

In this context, pragmatic diagnostic approaches are essential. For patients presenting with a medullary syndrome originating from endemic areas, the detection of *Schistosoma* spp. in CSF or evidence of systemic schistosomiasis should prompt early therapeutic intervention, even in the absence of definitive imaging.

This case highlights the diagnostic challenges of spinal schistosomiasis in endemic regions, where non-specific presentations overlap with other causes of myelopathy, including spinal tuberculosis, neoplastic lesions, and inflammatory or vascular disorders. Limited access to advanced imaging further contributes to diagnostic delay. Early antiparasitic treatment is associated with improved neurological and functional outcomes, whereas delayed diagnosis often results in persistent disability, as observed in our patient. From a public health perspective, strengthening schistosomiasis control programs, improving sanitation, and enhancing clinician and community awareness are essential measures to prevent transmission, promote early recognition, and reduce long-term neurological sequelae.

The main strengths of this study include the combination of a well-documented clinical case with a systematic synthesis of published evidence, providing epidemiological, clinical, and radiological insights into a neglected condition. Limitations include the retrospective nature of the reviewed cases, heterogeneity in reporting, and limited availability of long-term outcome data. Despite these limitations, our findings emphasize the importance of early recognition, improved diagnostic strategies, and heightened awareness of spinal schistosomiasis among clinicians working in endemic and non-endemic settings.

## 5 Conclusion

MS should be considered in young patients from endemic regions presenting with myelopathy. Early diagnosis and treatment are essential to prevent irreversible neurological sequelae. Further epidemiological studies and improved diagnostic strategies are essential to better understand and manage this severe form of schistosomiasis.

## Data Availability

The raw data supporting the conclusions of this article will be made available by the authors, without undue reservation.

## Footnotes

1 Preferred Reporting Items for Systematic Reviews and Meta-Analyses(PRISMA) flow diagram of selection and inclusion of studies in the systematic review.
