# Supplementary material for: Schistosoma infection as a cause of myeloradiculopathy: case report from Mozambique and systematic review
Source: Front Med (Lausanne). 2026 Feb 18;13:1725323. doi: 10.3389/fmed.2026.1725323 (PMC12956659; doi:10.3389/fmed.2026.1725323)
Supplement: Supplementary file 1 [file Table_1.pdf]

Order	Author, year	Nº of cases	Age	Sex	Country	Clinical features	Diagnosis	Local of lesion	Specie of Schistossoma	Treatment	Outcome
Current case	Buque et al 2026	1	27	M	Mozambique	Low back pain, spastic paraparesis, urinary incontinence,	MRI	T12-L4, conus medullaris		Praziquantel, steroids,	mRS 3-5<br>Paraparesis grade 3 in 4 months
1	Paubel et al., 2024(Paubel et al., 123 C.E.)	1	28	F	Kenya	flaccid paraplegia, Neurogenic blader, hypoesthesia, painful dysesthesia of lower limbs	MRI	T8 - conus medullaris	Mansoni	Praziquantel & Steroids & Gabapentin	mRS ≤ 2<br>Ataxic gait after 3 months
2	Abera et al., 2024(Abera et al., 2024)	2	12	M	Ethiopia	lower back pain, bilateral leg weakness, urinary retention, fecal incontinence.	MRI, stool microscopy	Conus medullaris	Mansoni	Praziquantel, steroids	mRS ≤ 2<br><br>MRI with Reduced oedema in 5 months
			14	M	Ethiopia	Low back pain, assimetric paraparesis, clonus	Urinary and stool microscopy	Dorsal spine	Mansoni	Praziquantel, steroids	mRS ≤ 2<br>Good clinical progress in 2 months
3	Sassi et al., 2024(Sassi et al., 2024)	1	13	M	Mauritânia	Cone medular syndrome: urinary retention and ataxic gate	Biopsy	Cone medullaris		Praziquantel, steroids	mRS ≤ 2<br>No sign of local recurrence in MRI
4	NyangWechi et al., 2024(*Conus Medularis Neuroschistosomiasis in a 12-Year Old Boy | East African Journal of*	1	12	M	Kenya	Flacid paraplegia grade 0	RMI	Cauda equina		Praziquantel	mRS =3-5<br>Some clinical improvement, with paralysis.

	Neurological Sciences, n.d.)										
5	Mohandas et al., 2023(Mohandas et al., 2023)	1	5	M	South Sudan	Paraparesis grade 2, urinany incontinence	MRI and Biopsy	Conus medull aris D11 – L1		Praziquantel and steroids	mRS = 3-5<br>Partial recovery
6	Mansour et al., 2023(Mansour et al., 2023)	1	29	M	Sudan	Flacid Paraparesis grade 3, urinary incontinence	MRI and biopsy	Toracic spine and Conus medull aris	Eggs of S. Mansoni	Praziquantel and steroids	mRS ≤ 2<br>(Complete resolution after 6 months)
7	Alayafi et al., 2022(Alayafi et al., 2022)	1	25	M	Saudi Arabia	Transverse myelitis: spastic paraplegic, urinary and bowel sphincter impairment, sensory level at T6	MRI<br><br>CSF high protein<br><br>Biopsy	Extensi ve (cervic odorsal) longitu dinal transve rse myeliti s	S. Mansoni eggs	Praziquantel and steroids	mRS=3-5<br>Partial improvement
8	Kollapen et al., 2022(Kollapen et al., 2021)	1	9	M	South Africa	Paraparesis grade 4/5	MRI<br><br>ELISA<br><br>Biopsy	Lumbar and conus medull aris (T10 – L1)	Bilharzia ova granulom as		
9	Brandao et al., 2021(De Barros Brandão et al., 2021)	1	11	M	Brasil	Paraparesis, urinary retention	Stool microscopy & MRI	Connus medull aris		Albendazol<br>Praziquantel<br>Steroids	mRS ≤ 2 (Total recovery)

10	Chew, R, et al., 2021(Chew, 2021)	1	31	M	Australia (travel from Uganda)	Low back pain, Paraparesis, fecal and urinary incontinence, neuropatic pain	MRI	Dorsal T8 to conus medull aris		Praziquantel and steroids	mRS= 3-5 Paraplegic, doubly incontinent and neuropatic pain after 4 years.
11	Dastoli et al.,2021(Dastoli et al., 2021)	1	13	F	Brasil	Lumbar pain, transverse myelitis: paraplegia grade 0, urinary and fecal incontinence, hypoesthesia and apalesthesia at T10	MRI histology	T4 – T10	S. mansoni eggs	Praziquantel and steroids, surgery	mRS=3-5 Improvement of pain and sensibility
12	Wilton et al.,2021(de Wilton et al., 2021)	4	50 (1)	M	UK (Nigerian travel 1 year before & swimmin g)	Transverse myelitis. Paraplegia and urinary and fecal incontinence	MRI ELISA	T4 – T10 and conus medull ar		Praziquantel, steroids, neurorehabilit ation	mRS=3-5 Remain Gait and urinary dysfunction, lumbar pain
			21 (2)	F	UK (travel to Uganda and Thailand	Paraplegia, fecal and urinary disfunction	CSF & serum serology, MRI	T7 – T8 and conus medull aris		Praziquantel, steroids	mRS ≤ 2 Complete recovery
			31 (3)	M	UK (travelled to Malawia nd swiiming at lake)	Lower limb pain, paresthesia , disuria	MRI Serum and CSF serology (ELISA)			Praziquantel, steroids	mRS ≤ 2 (Disesthesia and constipation)
			25 (4)	M	UK (traveled to	Lower limb Pain, paresthesia and urinary retention	MRI, CSF ELISA	T7 – T11		Praziquantel, steroids	mRS 3-5 (Ongoing urinary retention, paraparesis grade +4)

					Uganda & canoed in Lake Victoria)						
13	Domingues at al., 2020(Domingues et al., 2020)	2	36 (1)	M	Brazil	Assimetric spastic paraparesis grade 3/-3, Babinski sign, achiles clonus, sensitive level at T6, urinary retention	MRI<br>Stoll exam<br>Abdominal ltrasound	T6 – T10		Steroids, Praziquantel	mRS ≤ 2<br>Partial improvement, paraparesis grade 4
			37(2)	M	Brazil	Proximal assimetric spastic paraparesis grade ¾, hyposthesia lumbo-sacral L4-S1)	MRI<br>CSF	Anterior lumbar spine	s. mansoni	Steroids , praziquantel, Pregabalin (neuropatic pain)	mRS ≤ 2<br>Pestist hypoesthesia lumbosacral
14	Pambe et al.,2020(Pambe et al., 2020)	1	6	F	Cameroon	Medular syndrome	MRI<br>biopsy	T11-T12		praziquantel	mRS ≤ 2<br>Total recovery
15	Rodrigues et al., 2020(Rodrigues et al., 2020)	1	4	M	Brazil	Paraparesis grade 3, fecal incontinence, urinary retention.	MRI	T9-T12: conus medullaris	s. mansoni	Praziquantel	mRS ≤ 2<br>Paraparesis grade 4
16	Oliveira et al., 2020(de Oliveira et al., 2020)	1	11	M	Brazil	Acute Flacid Tetraparesis grade 4 upper/0 lower limb, C6 sensitive level, urinanry retention	RMI<br>ELISA for CSF	C6		Praziquantel, steroids,)	T mRS = 3 - 5<br>Tetraparesis grade 4, urinary catheter after 2 months
17	Silva et al., 2019(da Silva et al., 2019)	1	22	F	Brazil	Lumbar pain, radicular pain to right lower limb, acute paresthesia	MRI<br>ELISA	Conus medullar	S. Mansoni	Praziquantel, steroids, Gabapentin	mRS ≤ 2<br>Intermittent low-intensity pain
18	Bonnefond et al., 2018(Bonnefond et al., 2019)	1	63	M	France (travelled to Ivory Coast)	Acute febril eosinophilia, lower limb paresthesia,	MRI<br>ELISA	T2 – T8		Steroids, praziquantel (2 rounds)	mRS = 3 – 5<br>Paraplegia

						rapid and progressive paraplegia					
19	Al-Abdulwahhab et al., 2018(Al-Abdulwahhab et al., 2018)	1	13	M	Saudi Arabia	Back pain, paraparesis grade 3/5, hypoesthesia at lower limb.	MRI Serology	Thoracolumbar		Praziquantel	mRS ≤ 2 Total recovery
20	Detzler et al.,2018(Detzler et al., 2018)	1	27	M	Germany (from Eritrea)	Painful radiculopathy	MRI	T11 to medullary conus	S. Mansoni	Praziquantel 3 days, steroids 3 months	mRS ≤ 2 Total recovery
21	Machiels et al.,2018(Machiels et al., 2018)	1	17	M	Netherland (from Eritrea)	Back pain, progressive paraparesis	MRI, Serology, stool and CSF	Thoracolumbar	S. Mansoni	Praziquantel, Steroids	mRS ≤ 2 Total recovery
22	Boubacar et al., 2016(Boubacar et al., 2016)	1	25	M	Senegal	Acute progressive myeloradiculitis, genitosphincter dysfunction	Serology, eggs in urine, CSF hyperproteinorachia		S. Haematobium	Praziquantel, Steroids and physiotherapy	mRS ≤ 2 Partial recovery after 6 weeks
23	Salgado et al., 2015(Salgado et al., 2015)	1	6	M	Brazil	Holocranial headache, fever, parestesia, paraparesis and urinary incontinence	MRI, Stoll examination, CSF	Spinal cord	S. Mansoni	Praziquantel, pulsetherapy and long sterois course	mRS ≤ 2 Partial recovery,<br><br>Paraparesis after 2 years
24	Palin et al., 2015(Palin et al., 2015)	1	20	F	UK (from Eritrea)	Pregnant with progressive low back pain radiating to he buttock, paraparesis grade 4, saddle anaesthesia	MRI, histology	T10 - L2	S. Mansoni	Praziquantel and steroids, surgery	mRS ≤ 2 Total recovery after 6 months
25	Lia et al., 2014(Pappamikail et al., 2014)	1	38	M	Portugal (From Ivory Coast)	Progressive paraparesis, neurogenic bladder, hypotonia, sensory level.	MRI, Histopathistology	T4-T5	S. Mansoni	Praziquantel and steroids, surgery	mRS ≤ 2 Urinary catheterization after 6 months

26	Algahtani et al., 2014(Algahtani et al., 2014)	1		M	Saudi Arabia	Progressive paraparesis, urinary incontinence	MRI, Rectal biopsy	Conus medull aris	S. Mansoni	Praziquantel, steroids	
27	Harter et al., 2014(Härter et al., 2014)	1	16	M	German (from Ghana)	Obstipation, dysuria, dysaesthesia of the gluteal and lower limbs, plantar hypaesthesia and paraesthesia	CSF – DNA detension		Schistoso ma spp.	Praziquantel, steroids	mRS ≤ 2<br>Total recovery
28	Mikulich et al., 2013(Mikulich et al., 2013)	1	28	M	Ireland (From Sudan)	Severe low back pain for 5 years, flaccid paraplegia, sensory level T8 and urinary retention	MRI, CSF antiboby	T6 to conus medull aris	Schistoso ma ssp.	Praziquantel, steroids	mRS ≤ 2<br>Partial recovery
29	Jaber et al., 2013(Jaber & Kirby, 2013)	1			US (from Liberia)	Chronic hepatitis C with paraparesis	Spinal cord autopsy		S. Mansoni		mRS 6
30	Cisse et al., 2012(Cisse et al., 2012)	1	26	M	Senegal	Spastic paraparesis, acute urinary retention, low back pain and limbs	MRI, CSF and fluid serology	T11 – L1	S. mansoni	Praziquantel , steroids	mRS ≤ 2<br>Favourably in 2 years
31	Salim et al., 2012(Salim et al., 2012)	5	9 - 45	4M 1F	Sudan	Paraparesis, urinary incontinence, lower limb impaired sensation	Stools analisis, MRI	T12 – L2	S. mansoni	Praziquantel, steroids	mRS ≤ 2<br>1Total recovery<br>4 partial recovery
32	Badr et al., 2021(Badr et al., 2011)	17	13 - 30	M1 3 F4	Egypt		CSF serology, MRI		S. mansoni		7- total recovery (mRS ≤ 2)<br>8 – partial recovery (mRS ≤ 2)<br>2 – No response (mRS=3-5)
33	Crowell et al., 2011(Crowell et al., 2011)	1	6	M	Kenya	Acute low back pain, nonprogressive paraparesis	MRI,	T9 – L2, histolo gy	S. haemato bium	Praziquantel, steroids, physiotherapy	mRS ≤ 2<br><br>Total recovery after 3 months
34	Lobo et al., 2011(Lobo et al., 2011)	1	18	F	Portugal (from Brazil)	Acute distal flaccid paraparesis, hypoesthesia at L4 – S2 sensory level,	MRI, CSF, Serology	Conus medula ris	S. mansoni	Praziquantel, steroids	mRS ≤ 2<br>Total recovery after 6 months

						dysesthesia and hyperesthesia at lower limbs					
35	Jongste et al., 2009(de Jongste et al., 2010)	1	35	M	Netherlands (from Brazil)	Hypoesthesia in lower limbs, flaccid paraesthesia, urine retention	MRI, Serology	T12-L1	S. mansoni	praziquantel	mRS ≤ 2<br>Total recovery
36	Macdonald et al., 2009(Macdonald et al., 2009)	1	34	F	New Zeland (from South Africa)	Acute urinary retention, lower legs paraesthesia	MRI, stool microscopy	Conus medullaris	S. mansoni	Praziquantel, steroids	mRS ≤ 2<br>Total recovery
37	Poirier et al., 2009(Poirier et al., 2009)	1	29	M	France (travelled to Mali)	Low back pain, dysesthesia, difficulty in walking	MRI	T2-T8 and Conus medullary	S. mansoni	Praziquantel, Sterois	
38	Lighter et al., 2008(Lighter et al., 2008)	1	14	M	New York (travelling from Guinea)	Low back pain, mild weakness in lower limbs	MRI, CSF Serology	Conus medullaris	S. mansoni	Praziquantel, steroids	mRS ≤ 2<br>Total recovery
39	Ahmed et al., 2008(Ahmed et al., 2008)	1	44	M	Sudan	Diarrhoea, loss of weight, urinary retention flaccid paraparesis	MRI, rectal biopsy	Cauda equina	S. mansoni	Praziquantel, steroids	mRS = 3-5<br>Mild recovery, with Paraparesis
40	Jiang et al., 2008(Jiang et al., 2008)	4	36	M	China	Hyperesthesia at level of T9-L2, anaesthesia, paraparesis grade 0-1	CSF IgG, MRI, histology	T9 – L2	S. japonicum	Praziquantel, steroids, surgery	mRS ≤ 2<br>Partial recovery in1 year
			32	F	China	Progressive lower limbs pain, urinary and defecation difficulty, paraparesis grade 3-4	CSF IgG, MRI	Conus medullaris	S. japonicum	Praziquantel, steroids, surgery	mRS ≤ 2<br>Total recovery

			28	M	China	Pain and legs anaesthesia, flaccid paraparesis grade 2-3	CSF IgG (ELISA), MRI, histology	Conus medullaris	S. japonicum	Praziquantel, steroids, surgery	mRS ≤ 2<br>Partial recovery, with paraparesis grade 4-5 in 1 year
			40	M	China	Dysuria, low back pain, paraparesis grade 2, perianal hypoesthesia	CSF IgG (ELISA), MRI, histology	T11 – L1	S. japonicum	Praziquantel, steroids, surgery	mRS ≤ 2<br>Total recovery
41	Filho et al., 2007(Andrade Filho et al., 2007)	2	32	F	Brazil	Flaccid paraparesis, intestinal obstipation, urinary retention	CSF antiboby, MRI	T11-T12			mRS ≤ 2<br>Partial recovery
			32	M	Brazil	Flaccid paraparesis, urinary retention	MRI, Biopsy		s. mansoni	Praziquantel, steroids	
42	Wichmann et al., 2006(Wichmann et al., 2006)	1	24	M		sensation of cold legs and lumbago radiating to both soles, Lasegue sign	MRI	T12, conus medullaris		Praziquantel, sterois	mRS ≤ 2<br>Total recovery
43	Chen et al., 2006(Chen et al., 2006)	1	30	M		Lethargy, low back pain,lower limbs paresthesia ,constipation, urinary retention, flaccid paraparesis	MRI	Thoracic		Praziquantel, steroids	mRS ≤ 2<br>Total recovery after 15 months
44	Kim et al., 2006(Kim et al., 2006)	1	25	M	Brazil	Progressive subacute paraparesis, sensory deficits, urinary disturbance	MRI, CSF serology	Conus medullaris	S. mansoni		
45	Artal et al., 2006(Artal et al., 2006)	1	65	M	Brazil	Progressive paraparesis, urinary and fecal dysfuntion, lombossacral pain and tingling, lower limb hypo-palestesia, seizures,fasciobraquial hemiparesis	MRI, serology, biopsy	T6 to conus medullaris	S. mansoni	Praziquantel, steroids	mRS ≤ 2<br>Partial recovery

46	Saleem et al., 2005(Saleem et al., 2005)	8	9 – 42 (mean 21)	M	Sudan	Paraparesis, urinary incontinence, lower limb hypoesthesia	MRI, stools microscopy,	T12 – L2	S. mansoni	Praziquantel, steroid	mRS ≤ 2<br>One total recovery<br>4 partial recoveries
47	Camargos et al., 2005(Camargos et al., 2005)	1	46	M	Brazil	Progressive spastic paraparesis grade 4, hypoesthesia , hypopalestesia	MRI	T4- T8		Praziquantel, steroids	mRS ≤ 2<br>Total recovery
48	Pessoa et al., 2004(Pessoa et al., 2004)	1			Brazil	Myeloradiculopathy, intestinal obstruction	MRI, CSF	Conus medularris		Praziquantel, steroids	mRS ≤ 2<br>Total recovery
49	Artal and Vargas, 2004(Carod-Artal & Vargas, 2004)	2	28	F	Brazil	Paraparesis, hypoesthesia at sensory level of L5 – S1	MRI, CSF serology	Conus medullaris	S. mansoni	Praziquantel, steroids	mRS ≤ 2<br>Total recovery
			32	F	Brazil	Lumbosacral dysesthesia, myoclonic movements in lower limbs	MRI, serology	Conus medullaris	S. mansoni	Praziquantel, steroids	mRS ≤ 2<br>Total recovery
50	Labeodan and Sur, 2003(Labeodan & Sur, 2003)	1	10	M		Ascending paraparesis, sphincter dysfunction	MRI	Conus medullaris		Praziquantel, steroids	mRS ≤ 2<br>Total recovery
51	Koul et al., 2002(Koul et al., 2002)	1	8	M		Progressive ascending paraparesis, bowel and bladder incontinence	MRI, CSF serology, stools microscopy	Conus medullaris	S. mansoni	Praziquantel, steroids	
52	Silva et al., 2002(Silva et al., 2002)	1	34	M	Brazil	Cervical pain, Tetra paresis, tetraparestesia, hypoesthesia	MRI, byopsy and histology	C1 – C7	S. mansoni	Oxamniquine, surgery	mRS ≤ 2<br>Total recovery, residual parestesia

53	Junker et al, 2001(Junker et al., 2001)	1	40	M	Nigeria	Leg and arms pain and weakness, paraparesis, abdominal muscles paresis of C7-C8, hyperesthesia of T8-T12, tetraparesis	MRI, Serology	C6-C8	S. mansoni	Praziquantel, steroids	mRS ≤ 2<br>Partial recovery, spastic tetraparesis, able to walk
54	Owor et al., 2001(Owor et al., 2001)	2	18	F	Uganda	Spastic paraparesis grade 0, urinary incontinence, thoracic sensory level of T12	CT myelogram, Biopsy	T12	S. haemato bium	Praziquantel, surgery, steroids	mRS ≤ 2<br>Total recovery in 1 year
			21	M	Uganda	Back pain, progressive paraparesis, hypoesthesia, urinary incontinence	CT Mielogram, byopsy	T12		Praziquantel, steroids, surgery	mRS ≤ 2<br>Partial recovery
55	Silva et al., 2001(Nobre et al., 2001)	23		17 M<br>6F	Brazil	Low back pain, paraparesis, urinary retention,	CT myelogram, MRI	Thorac olumba r		Praziquantel, steroids	mRS ≤ 2<br>5 total recoveries<br>18 Partial recoveries
56	Sanelli et al., 2001(Sanelli et al., 2001)	1	41	F	US	Paraparesis, low back pain	MRI, retal biopsy	T12 – conus medull aris		Praziquantel, steroids	mRS ≤ 2<br>Partial recovery
57	Leite et al., 2000(Leite et al., 2000)	1	2	M	Brazil	Flaccid Paraparesis, constipation, urinary dysfunction	MRI, stool analysis	T9 - L1	S. mansoni	Praziquantel, Oxamniquine, steroids, surgery	mRS ≤ 2<br>Partial recovery
58	Lima et al., 1999(Ferreira Lima et al., 1999)	1	20	M	Brazil	Paraesthesia, low back pain and paraparesis	CSF serology			Oxaminiquine, steroids	mRS ≤ 2<br>Total recovery in 5 days

References

Abera, M. T., Abdela, A. F., Yaynishet, Y. A., & Tefera, T. G. (2024). Arborized pattern of MRI enhancement in spinal cord schistosomiasis: A report of 2 successful case outcomes. *Radiology Case Reports*, *19*(10), 4190–4194. https://doi.org/10.1016/j.radcr.2024.06.060

Ahmed, A. F., Idris, A. S., Kareem, A. M., & Dawoud, T. A. (2008). Acute toxemic schistosomiasis complicated by acute flaccid paraplegia due to schistosomal myeloradiculopathy in Sudan. *Saudi Medical Journal*, *29*(5).

Al-Abdulwahhab, A. H., Al-Sharydah, A. M., Al-Suhibani, S. S., Al-Jubran, S. A., Al-Haidey, A. K., Al-Hifzi, A. I., & Al-Issawi, W. (2018). Neuroschistosomiasis mimicking lower back pain: Case report of a rare differential diagnosis in a pediatric patient. *Patient Safety in Surgery*, *12*(1). https://doi.org/10.1186/s13037-018-0175-z

Alayafi, H. A., Alruwaili, M., Aljumah, T. K., Alshehri, A., Alrasheed, D., Alanazi, M. F., AlRuwaili, R., Ali, N. H., Albarrak, A. M., AlRashdi, B. M., AlRashdi, B. M., & Taha, A. E. (2022). Mycoplasma pneumoniae and Schistosoma mansoni co-infection in a young patient with extensive longitudinal acute transverse myelitis. *Journal of Infection in Developing Countries*, *16*(12), 1933–1938. https://doi.org/10.3855/jidc.17023

Algahtani, H. A., Aldarmahi, A. A., Al-Rabia, M. W., & Baeesa, S. S. (2014). Acute paraplegia caused by Schistosoma mansoni. *Neurosciences*, *19*(1), 47–51.

Andrade Filho, A. S., Queiroz, A. C., Freire, A. C. C., Lima, L. C. S., Filho, C. A. S., Amado, I. N., Reis, M. G., Magalhães, I. F., & Carmo, T. M. A. (2007). Pseudotumoral form of neuroschistosomiasis: Report of three cases. *Brazilian Journal of Infectious Diseases*, *11*(4), 435–438. https://doi.org/10.1590/S1413-86702007000400014,

Artal, F. J. C., Mesquita, H. M., Gepp, R. D. A., Antunes, J. S., & Kalil, R. K. (2006). Brain involvement in a Schistosoma mansoni myelopathy patient. *Journal of Neurology, Neurosurgery, and Psychiatry*, *77*(4), 512. https://doi.org/10.1136/JNNP.2005.078014

Badr, H. I., Shaker, A. A., Mansour, M. A., Kasem, M. A., Zaher, A. A., Salama, H. H., & Safwat, M. I. (2011). Schistosomal myeloradiculopathy due to Schistosoma mansoni: Report on 17 cases from an endemic area. *Annals of Indian Academy of Neurology*, *14*(2), 107–110. https://doi.org/10.4103/0972-2327.82796

Bonnefond, S., Cnops, L., Duvignaud, A., Bottieau, E., Pistone, T., Clerinx, J., & Malvy, D. (2019). Early complicated schistosomiasis in a returning traveller: Key contribution of new molecular diagnostic methods. *International Journal of Infectious Diseases*, *79*, 72–74. https://doi.org/10.1016/j.ijid.2018.11.018

Boubacar, S., Diagne, N. S., Ben Adji, D. W., Diop, A. M., Seydi, M., Maiga, Y., Toure, K., Ndiaye, M., Diop, A. G., & Ndiaye, M. M. (2016). Myeloradiculitis due to Schistosoma haematobium: about an observation in Dakar (Senegal) | Myéloradiculite à Schistosoma haematobium : à propos d'une observation à Dakar (Sénégal). *Bulletin de La Societe de Pathologie Exotique*, *109*(2), 77–79. https://doi.org/10.1007/s13149-016-0479-4

Camargos, S. T., Dantas, F. R., & Teixeira, A. L. (2005). Schistosomal myelopathy mimicking spinal cord neoplasm. *Scandinavian Journal of Infectious Diseases*, *37*(5), 365–367. https://doi.org/10.1080/00365540510035337

Carod-Artal, F. J., & Vargas, A. P. (2004). Myelopathy due to Schistosoma mansoni. A description of two cases and review of the literature | Mielopatía por Schistosoma mansoni. Descripción de dos casos y revisión de la bibliografía. *Revista de Neurologia*, *39*(2), 137–141. https://doi.org/10.33588/rn.3902.2003095

Chen, A. W. Y., Alam, M. H., Williamson, J. M. L., & Brawn, L. A. (2006). An unusually late presentation of neuroschistosomiasis. *Journal of Infection*, *53*(3). https://doi.org/10.1016/j.jinf.2005.11.003
Chew, R. (2021). Probable spinal neuroschistosomiasis manifesting as transverse myelitis. *American Journal of Tropical Medicine and Hygiene*, *105*(6), 1439–1441. https://doi.org/10.4269/ajtmh.21-0649
Cisse, F. A., Morel, Y., Bangoura, M. A., Jedou, A., Basse, A., Ndiaye, M., Diop, A. G., Ndiaye, M. M., & Cisse, A. (2012). Myelopathy due to Schistosoma mansoni | Myélopathie à Schistosoma mansoni. *Revue de Medecine Interne*, *33*(10), 580–582. https://doi.org/10.1016/j.revmed.2012.06.005
Conus Medularis Neuroschistosomiasis in a 12-year old boy | East African Journal of Neurological Sciences. (n.d.). Retrieved August 16, 2025, from https://theeajns.org/index.php/eajns/article/view/214
Crowell, C., Kiruga, J. M., Figaji, A., Simat, K., Padayachy, L., Pillay, K., & Yogev, R. (2011). Neuroschistosomiasis due to schistosoma haematobium presenting as spinal cord tumor. *Pediatric Infectious Disease Journal*, *30*(11), 1006–1008. https://doi.org/10.1097/INF.0b013e31822769bd
da Silva, M. A., Nai, G. A., Tashima, N. T., Chagas, F. N., Basso, S. M., Geraldini, A. C. F., Marques, R. J., & Rocha, T. D. C. (2019). Schistosomal myeloradiculopathy - A case report. *Revista Da Sociedade Brasileira de Medicina Tropical*, *52*. https://doi.org/10.1590/0037-8682-0335-2018,
Dastoli, P. A., Leite, A. L., da Costa, M. D. S., Nicácio, J. M., Pinho, R. S., Ferrarini, M. A. G., & Cavalheiro, S. (2021). Medullary neuroschistosomiasis in adolescence: case report and literature review. *Child's Nervous System*, *37*(9), 2735–2741. https://doi.org/10.1007/s00381-021-05267-9
De Barros Brandão, L. H. G., De Melo Cavalcanti, C. N., Fachin, L. P., Moura, A. Á., & De Carvalho Ruela Pires, A. C. (2021). Schistosomal Myeloradiculopathy: Case report in a pediatric patient. *Medicina (Brazil)*, *54*(3). https://doi.org/10.11606/issn.2176-7262.rmrp.2021.172776
de Jongste, A. H. C., Tilanus, A. M. R., Bax, H., Willems, M. H. A., van der Feltz, M., & van Hellemond, J. J. (2010). New insights in diagnosing Schistosoma myelopathy. *Journal of Infection*, *60*(3), 244–247. https://doi.org/10.1016/j.jinf.2009.12.002
de Oliveira, L. S., de Sio Puetter Kuzma, G., Costa, L. C. V., & João, P. R. D. (2020). Schistosomal myeloradiculopathy in a non-endemic area | Mielorradiculopatia esquistossomótica em região não endêmica. *Revista Paulista de Pediatria*, *38*. https://doi.org/10.1590/1984-0462/2020/38/2018232
de Wilton, A., Aggarwal, D., Jäger, H. R., Manji, H., & Chiodini, P. L. (2021). Delayed diagnosis of spinal cord schistosomiasis in a non-endemic country: A tertiary referral centre experience. *PLoS Neglected Tropical Diseases*, *15*(2). https://doi.org/10.1371/journal.pntd.0009161
Detzler, J., Backes, H., & Guldner, J. (2018). Radiculomyelopathy in schistosomiasis | Radikulomyelopathie bei Schistosomiasis. *Nervenarzt*, *89*(8), 928–933. https://doi.org/10.1007/s00115-017-0453-5
Domingues, A. L. C., Barbosa, C. S., Agt, T. F. A., Mota, A. B., Franco, C. M. R., Lopes, E. P., Loyo, R., & Gomes, E. C. S. (2020). Spinal neuroschistosomiasis caused by Schistoma mansoni: Cases reported in two brothers. *BMC Infectious Diseases*, *20*(1). https://doi.org/10.1186/s12879-020-05428-2
Ferreira Lima, W. L., dos Santos Ferreira Tose, C., & dos Santos Ferreira, R. E. (1999). Spinal Neuroschistosomiasis diagnosis after spinal anesthesia. Case report | Diagnostico de neuroesquistossomose medular apos anestesia subaracnoidea. Relato de Caso. *Revista Brasileira de Anestesiologia*, *49*(5), 338–340.

Härter, G., Frickmann, H., Zenk, S., Wichmann, D., Ammann, B., Kern, P., Fleischer, B., Tannich, E., & Poppert, S. (2014). Diagnosis of neuroschistosomiasis by antibody specificity index and semi-quantitative real-time PCR from cerebrospinal fluid and serum. *Journal of Medical Microbiology*, *63*(PART 2), 309–312. https://doi.org/10.1099/jmm.0.066142-0

Jaber, O. I., & Kirby, P. A. (2013). Spinal cord schistosomiasis: Unexpected postmortem finding. *American Journal of Clinical Pathology*, *140*(1), 33–36. https://doi.org/10.1309/AJCPQR0ELSAAWJHW

Jiang, Y. G., Zhang, M. M., & Xiang, J. (2008). Spinal cord schistosomiasis japonica: a report of 4 cases. *Surgical Neurology*, *69*(4), 392–397. https://doi.org/10.1016/j.surneu.2007.02.026

Junker, J., Eckardt, L., & Husstedt, I. (2001). Cervical intramedullar schistosomiasis as a rare cause of acute tetraparesis. In *Clinical Neurology and Neurosurgery* (Vol. 103). www.elsevier.com/locate/clineuro

Kim, A. H., Maher, C. O., & Smith, E. R. (2006). Lumbar intramedullary spinal schistosomiasis presenting as progressive paraparesis: Case report. *Neurosurgery*, *58*(5). https://doi.org/10.1227/01.NEU.0000210223.25400.C7,

Kollapen, K., Ebrahim Suleman, F., Smuts, I., & Siwela, L. (2021). Medullary neuroschistosomiasis in a pediatric patient: a case report. *Radiology Case Reports*, *17*(3), 462. https://doi.org/10.1016/J.RADCR.2021.11.018

Koul, R., Alexander, P., Scrimgeour, E., Idris, M., & Joseph, K. (2002). Schistosoma mansoni myeloradiculopathy in an 8-year-old Omani boy. *Journal of Tropical Pediatrics*, *48*(3), 183–186. https://doi.org/10.1093/tropej/48.3.183

Labeodan, O. A., & Sur, M. (2003). Intramedullary schistosomiasis. *Pediatric Neurosurgery*, *39*(1), 14–16. https://doi.org/10.1159/000070873

Leite, C. C., Souza, A. F., Valente, M., Araujo, M. A., & Jinkins, J. R. (2000). Clinics in diagnostic imaging (52). Spinal cord schistosomiasis. *Singapore Medical Journal*, *41*(8), 417–419.

Lighter, J., Kim, M., & Krasinski, K. (2008). Intramedullary Schistosomiasis Presenting in an Adolescent With Prolonged Intermittent Back Pain. *Pediatric Neurology*, *39*(1), 44–47. https://doi.org/10.1016/j.pediatrneurol.2008.03.016

Lobo, P. P., Coelho, M., Geraldes, R., Santos, C., Grácio, M., Rosa, M. M., & Antunes, J. L. (2011). Myeloradiculopathy associated to Schistosoma mansoni. *BMJ Case Reports*, *2011*, bcr1220103631. https://doi.org/10.1136/BCR.12.2010.3631

Macdonald, C. A., Jardine, D. L., Hurrell, M. A., & Pithie, A. D. (2009). The worm that turned. *Transactions of the Royal Society of Tropical Medicine and Hygiene*, *103*(10), 1065–1067. https://doi.org/10.1016/j.trstmh.2009.05.007

Machiels, J. D., Cobussen, M., Bosboom, R. W., Van Os, N. J. H., Hageman, A. T. M., & Hassing, R.-J. (2018). Myelitis transversa caused by neuroschistosomiasis | Myelitis transversa door schistosomiasis. *Nederlands Tijdschrift Voor Geneeskunde*, *162*(20).

Mansour, M. A., Bayoumi, M., Hamdi, A., Moawad, Y., Ayad, A. A., & Ahmadi, Z. (2023). Spinal schistosomiasis masquerading as an intramedullary tumor. *IDCases*, *32*. https://doi.org/10.1016/j.idcr.2023.e01759

Mikulich, O., Chaila, E., Crotty, J. M., & Watts, M. (2013). Spinal cord schistosomiasis presenting as a spinal cord syndrome. *BMJ Case Reports*. https://doi.org/10.1136/bcr-2013-200229

Mohandas, P., Sarkar, H., Jain, D., & Sundaram, V. K. G. (2023). A case report of conus intramedullary mansoni neuroschistosomiasis. *Surgical Neurology International*, *14*. https://doi.org/10.25259/SNI_47_2023

Nobre, V., Silva, L. C., Ribas, J. G., Rayes, A., Serufo, J., Lana-Peixoto, M., Marinho, R. F., Lambertucci, J., Sarah Kubistchek, H., das Pioneiras Sociais, A., & Horizonte, B. (2001). Schistosomal Myeloradiculopathy due to Schistosoma mansoni: Report on 23 Cases. *Mem Inst Oswaldo Cruz, Rio de Janeiro*, *96*, 137–141.

Owor, G., Korolev, A., Ssenyonjo, H., & Kiryabwire, J. (2001). Schistomiasis of the spinal cord: Report two cases. *East African Medical Journal*, *78*(1), 49–51. https://doi.org/10.4314/EAMJ.V78I1.9113,

Palin, M. S., Mathew, R., Towns, G., & Scott Palin, M. (2015). British Journal of Neurosurgery Spinal neuroschistosomiasis Spinal neuroschistosomiasis. *British Journal of Neurosurgery*, *29*(4), 582–584. https://doi.org/10.3109/02688697.2015.1016890

Pambe, C. J. R.-N., Ngaroua, D., Amvene, J. M., Kabeyene, A. C., & Nkodo, J. M. M. (2020). Histopathology of a rare case of intramedullary schistosomiasis and literature review | Histopathologie d´un rare cas de schistosomiase intramédullaire et revue de la littérature. *Pan African Medical Journal*, *37*, 1–5. https://doi.org/10.11604/pamj.2020.37.153.24890

Pappamikail, L., Fernandes, P., & Gonçalves, C. (2014). Medullary schistosomiasis. *Surgical Neurology International*, *5*(Supplement), 66. https://doi.org/10.4103/2152-7806.132235

Paubel, T., Schneider, V., Lenfant, M., Christelle, ·, & Labarre, B. (123 C.E.). A tropical myelitis. *Neurological Sciences*, *45*, 5517–5519. https://doi.org/10.1007/s10072-024-07743-x

Pessoa, F. S. R. D. P., Rolim, T. M. D. L., Leite, A. B., De Menezes, D. B., Neto, J. H. S., & Callado, M. R. M. (2004). Bowel obstruction due to schistosomotic pseudotumor associated to neuroschistosomiasis | Obstrução intestinal por pseudotumor esquistossomótico associado a neuroesquistossomose. *GED - Gastrenterologia Endoscopia Digestiva*, *23*(6), 279–281.

Poirier, P., Brugières, P., Liance, M., Hosseini, H., Perignon, A., & Botterel, F. (2009). Sciatica after return from Mali: Case report of schistosomal myelopathy | Sciatique au retour du Mali: Une observation de myélopathie bilharzienne. *Medecine Tropicale*, *69*(3), 295–297.

Rodrigues, D. B., Rodrigues, A. B., Queiroz, J. W. M., Braga, M. C., Kita, W. S., Netto, R. H. D., de Souza, R. W., Napoli, P. R., & de Luna, A. A. A. N. F. (2020). Intramedullary spinal schistosomiasis in a child with acute myelopathy: A case report. *Surgical Neurology International*, *11*. https://doi.org/10.25259/SNI_484_2020

Saleem, S., Belal, A. I., & El-Ghandour, N. M. (2005). Spinal cord schistosomiasis: MR imaging appearance with surgical and pathologic correlation. *American Journal of Neuroradiology*, *26*(7), 1646–1654.

Salgado, J. V., Da Silva Cruz Salgado, I. A., Braga Júnior, L. L., Marques Serra, S. C., De Carvalho Barros, V. M., Alves Silva, M. J., & Monteiro-Neto, V. (2015). Myeloradicular Form of Neuroschistosomiasis in a Six-Year-Old Boy Infected With Schistosoma mansoni. *The Pediatric Infectious Disease Journal*, *34*(12), 1409–1411. https://doi.org/10.1097/INF.0000000000000902

Salim, A. D., Arbab, M. A., El Hassan, L. A., & El Hassan, A. M. (2012). Schistosomiasis of the spinal cord_ report of 5 cases. *Eastern Mediterranean Health Journal*, *18*.

Sanelli, P. C., Lev, M. H., Gonzalez, R. G., & Schaefer, P. W. (2001). Unique linear and nodular MR enhancement pattern in schistosomiasis of the central nervous system: Report of three patients. *American Journal of Roentgenology*, *177*(6), 1471–1474. https://doi.org/10.2214/ajr.177.6.1771471

Sassi, S., Touarsa, F., Jaradat, T., Arkha, Y., El Ouazzani, H., & Cherradi, N. (2024). Intramedullary spinal schistosomiasis: A case report and review of the literature. *International Journal of Surgery Case Reports*, *122*. https://doi.org/10.1016/j.ijscr.2024.110103

Silva, L. C. S., Kill, C. M., & Lambertucci, J. R. (2002). Cervical spinal cord schistosomiasis. *Revista Da Sociedade Brasileira de Medicina Tropical*, *35*(5), 543–544. https://doi.org/10.1590/S0037-86822002000500023

Wichmann, D., Hofmann, C., Sudeck, H., Burchard, G.-D., & Moser, A. (2006). Myeloradiculitis: a Rare Event in Schistosoma Infection Case Report On Neurological Examination On Physical Examination. *Infection*, *34*, 349–351. https://doi.org/10.1007/s15010-006-5116-6
